# Risk Stratification of Differentiated Thyroid Cancer: A Single-Center Study in Basrah

**DOI:** 10.7759/cureus.47990

**Published:** 2023-10-30

**Authors:** Mahmod S Jasim, Ibrahim H Hussein, Haider A Alidrisi, Abbas A Mansour

**Affiliations:** 1 Diabetes and Endocrinology, Faiha Specialized Diabetes, Endocrine and Metabolism Center, Basrah, IRQ; 2 Diabetes and Endocrinology, University of Basrah, College of Medicine, Basrah, IRQ

**Keywords:** iraq, ata risk stratification, dynamic risk stratification, differentiated thyroid cancer, thyroid

## Abstract

Background

Differentiated thyroid cancer is a common endocrine cancer; most of it has an indolent course and favorable outcomes, with a subset of patients having the risk of disease recurrence, which can be assessed using the fixed American Thyroid Association (ATA) risk stratification system or the dynamic response to therapy risk stratification that can be modified during patients follow-up.

Aim

The aim of this article is to assess the risk stratification of patients having differentiated thyroid cancer.

Methods

This is a retrospective cross-sectional study in which we evaluated medical records of 75 patients having differentiated thyroid cancer to assess the baseline ATA risk of recurrence and compared it to the results of dynamic risk stratification in response to therapy at 6-12 months post-surgery and at the last visit. Thyroglobulin level, anti-thyroglobulin antibody, thyroid ultrasound, and cytopathological examination were used to determine dynamic response to therapy and divided subjects into four groups: excellent response (ER), biochemical incomplete response (BIR), structural incomplete response (SIR), and indeterminate response (IR).

Results

At baseline, 55 patients had low risk, 14 patients had intermediate risk, and six patients had high risk. At 6-12 months post-surgery, in the low-risk group, ER, BIR, and IR responses were observed in 56.4%, 5.5%, and 38.2% of patients, respectively, and none of them exhibited SIR. In the intermediate-risk group, ER, BIR, and IR responses were observed in 57.1%, 21.4%, and 21.4% of patients, respectively, and none exhibited SIR. Among the high-risk group, two patients had ER, two patients had BIR, one patient had IR, and one patient had SIR. At the last visit, ER, BIR, and IR were observed in 65.5%, 9.1%, and 25.5% of low-risk patients, respectively, and no patient developed SIR. In the intermediate-risk group, ER, BIR, and IR were observed in 50%, 21.4%, and 28.6% of patients, respectively, and no patients developed SIR. Among the high-risk group, three patients achieved ER, one had BIR, one had IR, and one had SIR.

Conclusion

Most of the differentiated thyroid cancers in this study are low-risk. Dynamic risk stratification appears to be an effective tool in the follow-up of this population of patients having differentiated thyroid cancer.

## Introduction

Thyroid cancer is one of the most prevalent endocrine malignancies that accounts for 3.4% of all malignancies over the world [1], of which papillary thyroid cancer (PTC) is the commonest histological type, followed by follicular thyroid cancer (FTC) [2].

The incidence of these tumors has been constantly increasing worldwide over the last few decades [3]. Despite the increasing incidence, the mortality of these slowly growing tumors remains low [3,4]. These tumors have good overall outcomes, with a 10-year survival rate of 93% for patients with papillary type (PTC) and 85% for patients with follicular type (FTC) [5].

Proper risk stratification of malignant disease is essential to avoid the overtreatment of low-risk patients and under-treatment of high-risk patients [5]. Patients older than 20 years and younger than 60 years have a higher risk of recurrence compared to the risk of mortality [6], which means that the risk of recurrence is much greater than the risk of disease-specific death, implying that systems that are made to assess mortality in differentiated thyroid cancer (DTC) will not help predict recurrence of disease [7,8]. Therefore, many systems have been proposed over the past years to assess the risk of recurrence of DTC and predict survival [9], such as the American Joint Committee on Cancer (AJCC)/Tumor Node Metastasis (TNM) staging system, which has been designed to predict disease-specific survival (DSS) [10,11], and the American Thyroid Association (ATA) Risk Stratification Systems, which have been used to measure the probability of recurrent disease [4]. This stratification system uses information available from histopathological results and findings during surgery, in addition to preoperative and early postoperative clinical data, to assess the likelihood of the presence of persistent/recurrent disease [4,12]. Accordingly, patients are divided into three risk categories, namely high, intermediate, and low-risk categories, which will determine if further actions are needed [13].

Even though both the AJCC/TNM staging system and the ATA risk-stratification system add enormous information concerning the initial risk stratification, they are both static tools and provide suboptimal prediction for single patient risk over a long-term basis [14]. This has led to the development of a new concept known as dynamic (modifiable) risk stratification, in which the initial risk values are continuously refined, modified, and adjusted with time (to account for the effect of treatment) and as new information becomes available during patient follow-up to provide more reliable risk estimate for that particular patient [13-15].

Dynamic risk stratification uses measurements of serum thyroglobulin (Tg) (stimulated or suppressed), anti-thyroglobulin (TgAb) antibodies, and the findings of imaging studies, such as thyroid ultrasonography and radioactive iodine (RAI) scintigraphy, to reclassify patient into one of four categories. The first category is excellent response (ER), the second one is biochemical incomplete response (BIR), the third one is structural incomplete response (SIR), and the last one is indeterminate response (IR) [4].

Aim of the study

The objective of this study is to assess the initial and dynamic risk stratification among patients having DTC in Basrah.

## Materials and methods

A retrospective cross-sectional study was conducted in the Faiha specialized Diabetes Endocrine and Metabolism Center (FDEMC) in Basrah. We retrieved the medical records of 195 patients from the medical electronic data system who were referred to FDEMC between January 2010 and April 2023. The included patients are primarily of two categories. The first category has patients who were diagnosed mainly, managed, and followed by endocrinologists in FDEMC. These patients were managed according to the latest ATA guidelines for managing thyroid cancer available for the respective year. The second category has patients who were diagnosed and managed (surgery ± neck dissection ± radioactive iodine [RAI] therapy) primarily in other healthcare facilities (general hospitals, private hospitals, and private clinics) by non-endocrinologists (general surgeons, internists, oncologists, and radiotherapists) and then referred to FDEMC to continue follow-up. Those patients were managed according to the treating doctor's experience in managing thyroid cancer.

We included patients with DTC who have the following conditions: age ≥ 18 years, PTC or follicular thyroid cancer, completed histopathological reports, and follow-up after primary treatment (surgery ± neck dissection ± RAI) for six months or more.

**Figure 1:**
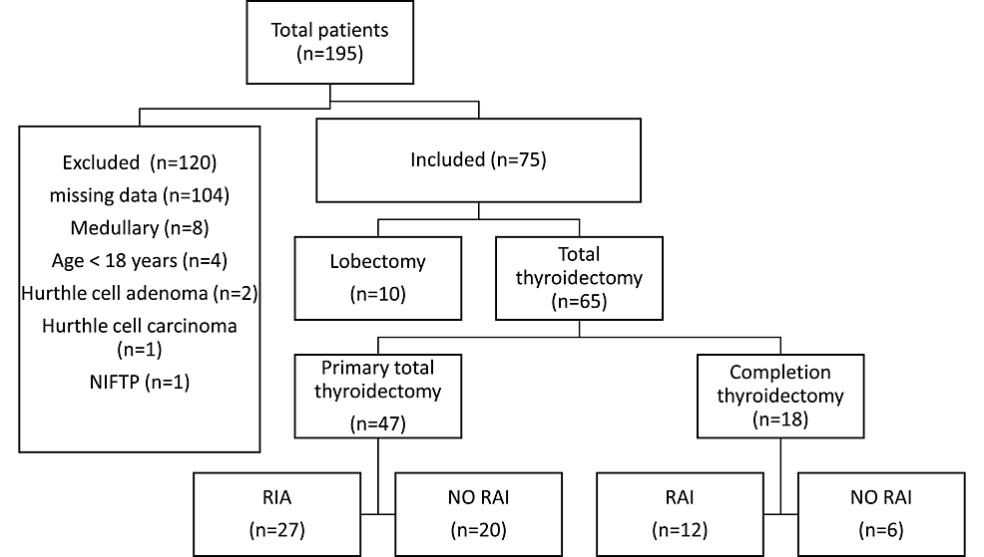
Schematic representation of studied patients NIFTP: Non-invasive follicular thyroid neoplasm with papillary-like nuclear features; RAI: Radioactive iodine therapy.

We excluded patients with the following conditions: medullary thyroid cancer, anaplastic thyroid carcinoma, thyroid lymphoma, lack of histopathological reports, and lack of follow-up for at least six months post-initial therapy. The total number of patients included and excluded as well as the type of primary treatment used in each patient are illustrated in Figure 1.

The initial risk stratification was done for all patients using the ATA Risk Stratification System, which divided patients into high, intermediate, and low-risk categories. The disease was staged according to the American Joint Committee on Cancer (AJCC)/TNM staging system, and patients were staged into four groups, stages I-IV, accordingly.

The modifiable risk stratification was applied to all patients using Tg level, TgAb, neck ultrasonography, and RAI scintigraphy (when available) during follow-up, which was designated to be at 6-12 months and the last visit after primary treatment (surgery ± neck dissection ± RAI ablation).

Biochemical analysis

In most patients, non-stimulated Tg was used (stimulated Tg level was used when available). These tests were conducted by Elecsys Tg II kit, Roche Cobas® e411, with a normal range of 1.40-78 ng/ml (lowest detection limit is 0.04 ng/ml). Anti-Tg antibodies were measured by Elecsys Anti-Tg Kit, Roche Cobas® e411, with a normal range of 10-115 IU/ml, and values > 115 IU/ml were considered positive for TgAb.

Imaging studies

A neck ultrasound was done using an Affiniti 50G Philips machine with an L12-4 probe by an endocrinologist specializing in thyroid ultrasound. RAI whole body scan (WBS) results were not available for all patients who had undergone RAI, and fluorodeoxyglucose-positron emission tomography (FDG-PET) scan was not obtained, so the decision about the presence of structural disease was primarily dependent on thyroid U/S as well as cytological and histopathological evidence of recurrent/persistent disease.

Radioactive iodine status

All patients in the high-risk group received at least one dose of radioactive iodine; 11 out of 14 patients in the intermediate-risk group received RAI, and 22 out of 55 patients in the low-risk group received RAI.

In this study, we used the following dynamic response to therapy definitions according to the type of primary treatment:

ER for total thyroidectomy plus RAI therapy: unstimulated Tg level below 0.2 ng/ml or stimulated Tg level below one ng/mL, negative TgAb, and negative imaging. ER for total thyroidectomy without RAI therapy: non-stimulated Tg < 0.2 ng/mL or stimulated Tg below two ng/mL, and undetectable TgAb plus negative imaging. ER for lobectomy: stable, non-stimulated Tg < 30 ng/mL, and undetectable TgAb plus negative imaging [12,16].

The definition of BIR for total thyroidectomy and RAI therapy: non-stimulated Tg level of more than one ng/mL, stimulated Tg level of more than 10 ng/mL, or increasing TgAb levels plus negative imaging. BIR for total thyroidectomy without RAI therapy: non-stimulated Tg > five ng/mL, stimulated Tg more than 10 ng/mL, increased Tg values over time with similar TSH levels, or increasing TgAb levels plus negative imaging. BIR for lobectomy: non-stimulated Tg > 30 ng/mL, increasing Tg values over time with similar TSH levels, or increasing TgAb levels plus negative imaging [12,16].

The definition of IR for total thyroidectomy with RAI therapy: unstimulated Tg level 0.2-1 ng/mL, stimulated Tg level 1-10 ng/mL, stable or decreasing TgAb levels, and no definite specific imaging findings. IR for total thyroidectomy without RAI therapy: no specific imaging findings, faint uptake in thyroid bed on RAI scanning, non-stimulated Tg of 0.2-5 ng/mL, stimulated Tg of 2-10 ng/mL, and stable or declining TgAb levels with no functional or structural evidence of disease. IR for lobectomy: no specific imaging findings, and stable or decreasing TgAb levels with no functional or structural evidence of disease [12,16].

SIR is defined as any functional or structural evidence of disease [12,16].

Statistical analysis

Statistical analysis was done by SPSS version 26 (IBM Corp., Armonk, NY). Mean, median, standard deviation, frequency, and percentage were utilized for data description. The chi-square test was used to determine the relationship between variables.

## Results

The data of 75 patients were included, and the basic characteristics of those patients are represented in Table 1. The mean age was 40.8 ± 10.6 years. DTC was more common in women than men, 61 versus 14 patients (81.3% vs 18.7%), respectively. Mean BMI was 30.3 ± 5.6 (kg/m^2^). About 91% of cases had PTC (n = 68), and about 93% of patients were in stage I (n = 69). According to ATA recurrence risk, about 73% of cases were in the low-risk group (n = 55), about 19% had intermediate risk (n = 14), and 8% had high risk (n = 6). Regarding RAI treatment, 52% of all cases received RAI (n = 39); the median cumulative dose was 100 mCi, with a range of 30-450. The median duration of follow-up was 30 months, with a range of 6-97. A family history of thyroid cancer was detected in two patients (2.7%) only.

**Table 1:** Basic characteristics of the enrolled patients having differentiated thyroid cancer ATA: American Thyroid Association; BMI: Body mass index; mCi: Millicurie; RAI: Radioactive iodine.

Variables	Mean ± standard deviation	Median (range)	Number (%)
Age at diagnosis (years)	40.8 ± 10.6	40 (24-68)	
Gender			
Male			14 (18.7)
Female			61 (81.3)
BMI (kg/m^2^)	30.3 ± 5.6		
Histology			
Papillary			68 (90.7)
Follicular			7 (9.3)
TNM staging			
Stage I			69 (93.3)
Stage II			4 (5.3)
Stage IVb			1 (1.3)
ATA recurrence risk			
Low risk			55 (73.3)
Intermediate risk			14 (18.7)
High risk			6 (8)
RAI (yes/no)			39 (52)/36 (48)
Cumulative dose of RAI, mCi	134.7 ± 105.4	100 (30-450)	
Duration of follow-up (months)	32.9 ± 23.5	30 (6-97)	
Family history of thyroid cancer			2 (2.7)

Results of dynamic risk stratification at 6-12 months post-surgery and the last visit in the three groups of initial ATA risk of recurrence are illustrated in Table 2. The dynamic response to treatment in the first 6-12 months after surgery shows the following outcomes: In the low-risk group (n = 55), 56.4% (n = 31) of cases achieved ER, 5.5% (n = 3) of cases had BIR, 38.2% (n = 21) had IR, and no patient had SIR. In the intermediate-risk group (n = 14), 57.1% of cases (n = 8) exhibited ER, 21.4% of cases (n = 3) were assigned to IBR, 21.4% of them (n = 3) had IR, and none of them exhibited SIR. Among high-risk patients (n = 6), two patients achieved ER, two patients showed BIR, one had IR, and one of them had SIR.

**Table 2:** Dynamic response at 6-12 months post-surgery and at the last visit in the three risk groups ATA: American Thyroid Association; BIS: Biochemical incomplete response; ER: Excellent response; IR: Indeterminate response; SIR: Structural incomplete response.

	Dynamic response at 6-12 months post-surgery				Dynamic response at last visit			
ATA risk group	ER	BIR	SIR	IR	ER	BIR	SIR	IR
Low risk (n = 55)	31 (56.4%)	3 (5.5%)	0	21 (38.2%)	36 (65.5%)	5 (9.1%)	0	14 (25.5%)
Intermediate risk (n = 14)	8 (57.1%)	3 (21.4%)	0	3 (21.4%)	7 (50%)	3 (21.4%)	0	4 (28.6%)
High risk (n = 6)	2 (33.3%)	2 (33.3%)	1 (16.7%)	1 (16.7%)	3 (50%)	1 (16.7%)	1 (16.7%)	1 (16.7%)

The dynamic response at the last visit shows the following: In the low-risk group (n = 55), 65.5% of patients (n = 36) had ER, 9.1% of them (n = 5) had BIR, 25.5% (n = 14) had IR, and none had SIR. Five patients previously assigned to IR were shifted to the ER, and two were moved to BIR.

Among the intermediate-risk group (n = 14), 50% of patients (n = 7) had ER, 21.4% (n = 3) exhibited BIR, 28.6% (n = 4) had IR, and none of them developed SIR. There is one patient who was previously classified to ER and was shifted to IR. Among high-risk patients (n = 6), three achieved ER, two had BIR, one had SIR, and one had IR. One patient who was previously assigned to BIR was shifted to the ER.

## Discussion

In this retrospective cross-sectional study, records of 75 patients with DTC were examined to assess risk stratification among patients having DTC in Basrah. Most DTCs were of papillary type and predominantly affected female patients; the majority were at stage I. These results are similar to the Jahanshahi et al.'s study conducted in Ahvaz, Iran [17]. The ATA recurrence risk is a validated tool to assess the risk of recurrence of DTC. Still, it was claimed that this tool is fixed and static and does not allow for the accommodation of recurrence risk following initial therapy [14].

Our study showed that the ATA risk of recurrence is likely to change with response to therapy in the first 6-12 months after surgery and at the last visit, in which all patients with ER, BIR, and IR did not develop SIR at the last visit regardless of the initial ATA risk. Half of the initially high-risk patients were shifted to the ER at the last visit. These results show that the baseline ATA risk can change with time.

Jahanshahi et al. conducted a cross-sectional study to describe the risk stratification of DTC in Ahvaz, Iran [17]. Their study found that risk stratification varied significantly based on dynamic risk, and when comparing with their results, they found none of the patients with high ATA risk achieved ER, whereas in our study, 3/6 patients achieved this result. This possibly can be explained by the fact that ongoing therapy with RAI might change risk category of patients having DTC. Also, in our study, none of the patients with intermediate ATA risk developed SIR, whereas in their study, 34% of the cases in this group had SIR. This finding can be explained by a difference in the sample size of the two studies.

Tuttle et al., in their study conducted in 2010, found that the use of dynamic risk stratification to predict the risk of recurrence is better than the ATA risk of recurrence during the first two years of follow-up [13].

The finding of this study differs from the finding of a study conducted by Mukhtar et al., in which 501 patients with DTC were included with a median follow-up of 101 months [18]. They found that 64.2% of patients having low ATA risk achieved ER at the initial evaluation, and this number increased to 95.1% at the last evaluation, whereas in our study, only 56.4% of patients in this category reached ER. This percentage increased to 65.5% at the end of follow-up; even more, they found that 16.1% of patients with intermediate ATA risk had SIR initially, which decreased to 10% at the last evaluation, whereas none of those patients in our study had SIR. Among patients with high ATA risk, 14.3% had ER at the first evaluation, which increased to 30.4% at the last evaluation, whereas in our study, 33.3% of subjects in this group had ER at 6-12 months after surgery and increased to 50% at the last visit. These differences might be attributable to a larger sample size, longer duration of follow-up, and difference in the standard of healthcare in Iraq compared to the Saudi healthcare system. Therefore, they concluded that the ATA risk of recurrence correlates well with short-term and long-term outcomes in DTC patients. In addition, they also found that most of the patients with IR and a good number of patients having BIR will achieve ER with time without the need for additional therapy [18].

In another study, Castagna et al., which included 512 patients with DTC [15], examined the performance of the static ATA risk of recurrence against dynamic risk assessment and found that half of the patients who were classified as intermediate to high ATA risk were moved to a low-risk category by dynamic risk assessment, and about one-tenth of patients having low ATA risk moved to a high-risk sort by dynamic risk assessment. They also found that the positive predictive value was 39.2% for ATA risk and 72.8% for dynamic risk; therefore, they concluded that dynamic risk is a better predictor of individual risk, making it a better choice to adjust subsequent therapy and follow-up [15]. These results are similar to the results of our study in which half of the patients with high ATA risk were moved to the ER at the end of follow-up, and no patient with low and intermediate risk developed SIR.

Momesso et al. studied 507 patients with DTC who were treated with lobectomy and total thyroidectomy without RAI. They found that among patients with ER, 0% developed recurrent/persistent disease, which is similar to our results. This concluded that dynamic risk stratification is an effective tool in the follow-up of patients managed with lobectomy and total thyroidectomy without receiving RAI. Their results supported less aggressive RAI use in patients with baseline intermediate risk [16]. They also found a 1.3% recurrence rate in the IR group and 31.6% in the BIR group, which none observed in our study. This may be because their patients were treated without RAI, while our patients received RAI.

Other studies confirmed the good outcomes of patients achieving ER. Hong et al., in their study to evaluate the performance of dynamic risk (named delayed risk stratification), found that about two-thirds of the ATA intermediate-risk group and one-fifth of the high-risk group were shifted to ER at the end of follow-up concluding that delayed risk stratification is better than ATA risk stratification in assessing the chance of structural disease progression [19].

Jeon et al. conducted a study on 1395 patients with ER to therapy; of them, 73% were labeled to have intermediate baseline ATA risk of recurrence and managed with total thyroidectomy plus RAI. They found that the recurrence rate among those patients was only 1% after a follow-up of 8.7 years, concluding the good prognosis among patients who achieved ER therapy [20].

Pitoia and Jerkovich reviewed several articles and found that ER at the end of follow-up was evident in 86%-91% of patients having low ATA risk, 57%-63% in the intermediate risk, and 14%-16% of patients with high risk [12].

The excellent prognosis in patients with ER is not the usual story. van Velsen et al., in a study, found a 14% recurrence rate in patients having ER in contrast to our results where none of our patients with ER developed recurrent/persistent disease. This is because van Velsen et al. studied only high-risk DTC with a high percentage of follicular thyroid cancer, and their patients were older than the patients enrolled in our study [9]. In concert with that, Tian et al. reported a 2.9% recurrence rate in patients having high ATA-risk PTC over a follow-up period of 5.6 years [21]. These findings suggest that patients with a high ATA risk of recurrence need more comprehensive and agile follow-up even if they achieve ER to initial therapy.

Other studies validated the performance of dynamic risk stratification. Park et al. validated the use of dynamic risk stratification in the follow-up of patients with DTC and found that the risk of recurrence varied significantly with response to treatment. They found that the hazard ratio for recurrence was 1.82 for IR, 20.8 for BIR, and 243.3 for SIR. The rate of ER among patients with low ATA risk was 72.7%, and no ER was observed in the high-risk group [22]. In addition, Abelleira et al. found a 0% recurrence rate in patients with ER to treatment, while there was 33.4% disease progression in patients with IR [23].

Limitations

This study has several limitations. This is a retrospective study with a small sample size and a short duration of follow-up. In addition, RAI WBS was unavailable to all patients due to limited access to them in Iraq. Furthermore, the lack of molecular studies and a shortage of FDG-PET scans in Iraq are also some limitations of this study.

## Conclusions

Most DTCs in this study are in the low-risk category, and PTC is the most frequent type. All patients with ER, BIR, and IR did not develop SIR at the end of follow-up. Patients with initially high ATA risk of recurrence can achieve ER with treatment but need a closer follow-up. Patients with SIR are unlikely to achieve ER. Dynamic risk stratification appears to be an effective tool in the follow-up of this population of patients having DTC.
